# The Viable Fabrication of Gas Separation Membrane Used by Reclaimed Rubber from Waste Tires

**DOI:** 10.3390/polym12112540

**Published:** 2020-10-30

**Authors:** Yu-Ting Lin, Guo-Liang Zhuang, Ming-Yen Wey, Hui-Hsin Tseng

**Affiliations:** 1 Department of Environmental Engineering, National Chung Hsing University, Taichung 402, Taiwan; a0938866458@gmail.com (Y.-T.L.); d102063002@smail.nchu.edu.tw (G.-L.Z.); 2 Department of Occupational Safety and Health, Chung Shan Medical University, Taichung 402, Taiwan; 3 Department of Occupational Medicine, Chung Shan Medical University Hospital, Taichung 402, Taiwan

**Keywords:** gas separation, thermally rearranged membrane, waste material, sustainable development, circular economy

## Abstract

Improper disposal and storage of waste tires poses a serious threat to the environment and human health. In light of the drawbacks of the current disposal methods for waste tires, the transformation of waste material into valuable membranes has received significant attention from industries and the academic field. This study proposes an efficient and sustainable method to utilize reclaimed rubber from waste tires after devulcanization, as a precursor for thermally rearranged (TR) membranes. The reclaimed rubber collected from local markets was characterized by thermogravimetric analyzer (TGA) and Fourier transfer infrared spectroscopy (FT-IR) analysis. The results revealed that the useable rubber in the as-received sample amounted to 57% and was classified as styrene–butadiene rubber, a type of synthetic rubber. Moreover, the gas separation measurements showed that the C7-P2.8-T250 membrane with the highest H_2_/CO_2_ selectivity of 4.0 and sufficient hydrogen permeance of 1124.61 GPU exhibited the Knudsen diffusion mechanism and crossed the Robeson trade-off limit. These findings demonstrate that reclaimed rubber is an appealing, cost effective, and sustainable alternative, as a precursor for TR membranes, for application in gas separation. The present approach is useful in the selection of a suitable reclaimed rubber precursor and related membrane preparation parameters, leading to the advancement in the recycling value of waste tires.

## 1. Introduction

Rapid population growth and industrialization are responsible for the significant increase in production and accumulation of solid waste. Advances in the automobile industry and increased use of vehicular modes of transport have led to an increase in the number of waste tires [1]. Currently, the global annual consumption of tires is up to 1 billion units [2,3]. Furthermore, the consumption rate of tires is expected to grow exponentially with their demand growing in developing countries. In Taiwan, the annual recovery market for waste tires is approximately 100 thousand tonnes, reaching approximately 140 thousand tonnes in 2017 according to the statistics of Environmental Protection Administration Executive Yuan, Republic of China (R.O.C.) (Taiwan) [4]. The most prevalent disposal methods for waste tires include landfilling and using them as auxiliary fuel for energy recovery [5,6]. However, landfilling is not the preferred option due to the large volumes of tires. Furthermore, the doughnut shape of tires retains stagnant water, which becomes a breeding habitat for mosquitoes, thereby resulting in the spread of vector-borne diseases [7], especially in humid environments found in countries like Taiwan. The incineration of waste tires emits pollutants including carbon monoxide (CO), carbon dioxide (CO_2_), sulfur oxides (SOx), toxic dioxins, polycyclic aromatic hydrocarbons, and particulate matter [8]. Therefore, the improper disposal and storage of waste tires can have adverse effects on the environment and human health. This necessitates the development of sustainable and efficient recovery and recycling methods for waste tire management. One of the best alternative solutions for dealing with waste tires is to transform them from waste material into valuable substances such as membranes. Considering the number of tires being discarded globally, rubber consumption is facilitating the exhaustion of limited petrochemical resources. Hence, in view of the circular economy and the drawbacks of current disposal methods for discarded tires, investigating technologies that can utilize waste materials are an inevitable tendency worldwide. For countries whose energy is imported, such as Taiwan, the reutilization of waste materials could boost their economic development because of the reduction in trade deficit. For example, the Taiwanese government is pressing ahead with the policy for a circular economy to provide financial support for companies and academic scientists to encourage and promote reutilization technology.

Recently, thermally rearranged (TR) membranes have been widely applied in the field of gas separation, such as hydrogen recovery, carbon dioxide capture, and natural gas purification, due to their unique microstructure and superior chemical/thermal stability in harsh environments [9,10]. TR membranes can be fabricated through a simple thermal rearrangement process after the heat treatment of a polymeric membrane, which facilitates the transformation of interconnected microcavities with a narrow cavity [11]. The thermal rearrangement process begins when the polymer aromatic chains undergoes a heating process by reaction of ortho-substituted atoms, creating a new ring. The buckling of the ring creates free volume elements in the membrane and enhances gas diffusion. Consequently, the microporous structure of TR membranes is obtained, which exhibits high gas permeance and great separation property. The frequently used polymer precursors for TR membranes mainly consist of polyimide, polybenzoxazoles, and polypyrrolone [10,11,12]. All the above-mentioned precursors are thermosetting polymers that are priced exorbitantly. The main constituent (approximately 47%) of the tires is rubber [13,14], usually synthetic, such as styrene–butadiene rubber and butadiene rubber [15]. All rubber materials are thermosetting polymers, except for thermoplastic elastomers. Thus, the rubber material in waste tires is viewed as a valuable resource, given its potential as a membrane precursor [16]. To date, no research has investigated tire-derived TR membranes for application in gas separation, which is likely restricted by their three-dimensional cross-linked structure, thereby resulting in inferior processability. Recently, methods for reclaiming rubber by physical, chemical, biotechnological, and de-link processes have been developed. The reclaimed rubber, with enhanced process flexibility, is converted from a three-dimensional cross-linked structure into a one-dimensional thermosetting polymer by destroying the rubber/sulfur bond [17]. This study was conducted using reclaimed rubber as the precursor for TR membranes. The effects of precursor concentration, incorporation of additives, and thermal treatment temperature on as-prepared TR membranes derived from reclaimed rubber were investigated.

### Research Significance

A huge amount of waste tires are produced worldwide due to the expansion of the automobile industry, and their disposal is an issue faced by all countries. The most common method to handle waste tires is landfilling or using them as auxiliary fuel for energy recovery. However, the published studies showed that scrapped rubber tires could not be decomposed under environmental conditions and pose a serious threat to the environment and human health. Moreover, the incineration of waste tires is accompanied with emissions of dioxins, volatile organic compounds and particulate matter. Consequently, recycling waste tire rubber by transforming it into a valuable material (e.g., membranes) has become the preferred solution to dispose of waste tires. From the circular economy perspective, reclaimed rubber-derived membrane is a reutilization alternative that contributed to waste reduction. Therefore, the goals of the present study were to provide a viable and green precursor (reclaimed rubber) with which to fabricate a TR membrane applied in the field of gas separation, thus boosting the circular economy via prolonging the life cycle of rubber tires. In order to further extend understanding of the reused rubber from waste tire, a series of characterizations were conducted to understand its characteristics and performance in related membranes. The membrane fabrication parameters, including precursor concentration, heating temperature, and the incorporation of a polyphenylene oxide (PPO) modifier, as well as the corresponding gas transport measurements, were also studied to produce an experimental evaluation of this new precursor for a gas separation membrane. To the best of our knowledge, there are no other existing studies on rubber-derived TR membranes applied in gas separation.

## 2. Experiment

### 2.1. Materials

**Figure 1:**
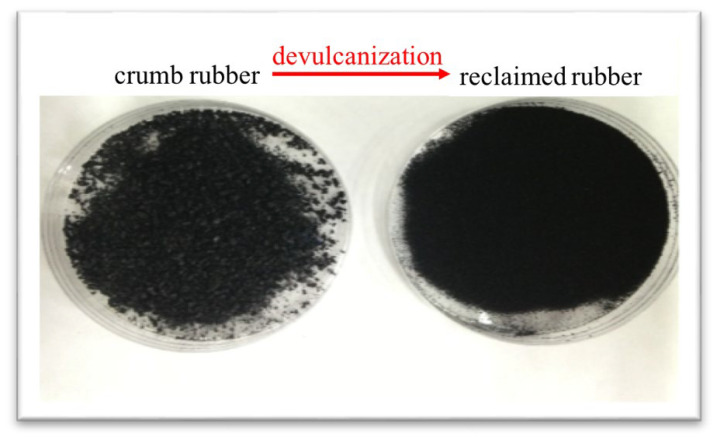
Optical image of as-received rubber sample provided from local market.

The crumb rubber sample and the reclaimed rubber sample selected as polymer precursors in this work were supplied by International Quality Materials Nano Tech. Co., Ltd. (Taipei City, Taiwan), as shown in Figure 1. Toluene (extra pure grade—99% purity, Union Chemical Works Ltd., Hsinchu City, Taiwan) was sourced as a solvent for the preparation of the casting solution. The polyphenylene oxide (PPO, Cas. No. 25134-01-4) purchased from Sigma-Aldrich (Saint Louis, MO, USA) was used as an additive to modify the microstructure of the TR membrane. Commercial ceramic embryos, with an average pore size of 74 Å, thickness of 1.4 mm, and diameter of 23 mm were obtained from Ganya Fine Ceramics Co. (New Taipei City, Taiwan). They were calcined at 1400 °C with a heating rate of 2 °C/min for 2 h holding time in our laboratory to enhance their physical strength. They were used as a bare substrate. The tested gases (99.999% pure)—H_2_, CO_2_, O_2_, N_2_, and CH_4_—were obtained from Toyo Gas Co. (Taichung City, Taiwan). All chemical reagents were used directly without purification.

### 2.2. Synthesis of Rubber-Derived TR Membrane

Initially, the different concentrations at 4, 5, 6, and 7 wt % of casting solutions were obtained by dissolving the desired amounts of collected reclaimed rubber in toluene solvents at a fixed temperature of 160 °C for 24 h. Similarly, casting solutions with various additive contents within the range of 0.7–2.8% were prepared. Afterwards, the as-prepared casting solution was applied onto an alumina substrate via the spin coating technique at a speed of 2000 rpm for 16 s to obtain the uniform rubber polymeric membrane. The fabricated rubber membranes were left overnight in an open environment at room temperature to allow the rest of the solvent in the membrane to evaporate. Subsequently, the two-stage thermal rearrangement procedure was performed in a tubular furnace (Chang-Hua Electric Heating Co., Ltd., Changhua city, Taiwan) in terms of a dried polymeric membrane. The polymeric membrane was placed in the central zone of a quartz tube equipped with a tubular furnace, and the heating environment was under a vacuum at less than 10^−6^ atm. The curing step was conducted at 200 °C with a ramping rate of 5 °C/min and then maintained for 6 h to stabilize the polymer chains. Next, the target temperatures (250 and 350 °C) were reached at the same heating rate (5 °C/min) and were maintained for 2 h. Finally, the TR membrane was naturally cooled to room temperature. To prevent aging, the pure gas permeation value of the resultant TR membrane was measured within 24 h after the conduction of the thermal rearrangement procedure. The TR membranes fabricated by different parameters were denoted as Cx-Py-Tz, where x is the precursor concentration, y is the content of PPO additive, and z is the heating temperature. For example, a C7-P0.7-T250 TR membrane means the polymeric membrane, with 7 wt % rubber precursor and 0.7 wt % PPO, was heated at 250 °C. Moreover, a C4-P0-T350 TR membrane refers to a polymeric membrane prepared with a 4 wt % rubber precursor, without the incorporation of PPO filler and heated at 350 °C.

### 2.3. Characterization of Collected Rubber Samples and Fabricated TR Membranes

The thermal behavior of the collected rubber samples from the recycling company was studied using a thermogravimetric analyzer (TGA, Perkin Elmer, simultaneous thermal analyzer 6000, Waltham, MA, USA). Prior to the analysis, the as-received rubber sample was placed in the oven at 60 °C for 24 h to remove any trapped moisture. The TGA testing of the rubber sample was conducted following the guidelines by Perkin Elmer [18]. To evaluate the polymer content in the sample, 6–8 mg of the dried sample was first heated in a nitrogen atmosphere, with temperatures increasing from 50–700 °C at the rate of 20 °C/min. Subsequently, the sample underwent second heating at 700–900 °C, at a fixed heating rate of 20 °C/min, in the air atmosphere to measure the carbon black content. The residue that remains undecomposed after heating until 900 °C was considered the other filler.

Fourier transfer infrared spectroscopy (FT-IR, FT/IR-4100 spectrophotometer, Jasco Corporation, Tokyo city, Japan) in the wavenumber range of 4000 to 400 cm^−1^ was performed to identify the functional group and to confirm the composition of the collected rubber sample.

The cross-linking density ($V_{e}$, mol/${\mathrm{cm}}^{3}$) of the rubber sample, presented in Equation (1), was obtained by combining the American Society for Testing and Materials (ASTM) D6814 standard and the Flory–Rehner equation [19,20].

(1) $$v_{e}=\frac{-[\ln\left(1-V_{r}\right)+V_{r}+X_{1}V_{r}^{2}}{\left[V_{1}\left(V_{r}^{\frac{1}{3}}-V_{r}\right)/2\right]}$$

where *V_r_* refers to the volume fraction of the swollen sample in the equilibrium with the pure solvent (toluene), $X_{1}$ refers to the polymer–solvent interaction parameter (0.446) [20], and $V_{1}$ refers to the molar volume of the toluene (106.35 ${\mathrm{cm}}^{3}$/mol).

The degree of devulcanization (%) of the as-received reclaimed rubber sample was evaluated using Equation (2):

(2) $$\mathrm{Devulcanization}\left(\%\right)=\frac{V_{r,reclaimed\;rubber\;}}{V_{r,crumb\;rubber}}\times 100\%$$

where *V_r,crumb rubber_* and *V_r,reclaimed rubber_* refer to the cross-linking densities of the rubber sample before and after the devulcanization process, respectively.

Scanning electron microscopy (SEM) micrographs of the rubber-derived TR membrane were obtained by a JEOL JSM-6700F scanning electron microscope (JEOL Ltd., Tokyo city, Japan) at an accelerating voltage of 3 kV to observe the membrane morphology. Prior to morphological analysis, the outer surface of each sample was sputtered with gold to improve its conductivity.

Atomic force microscopy (AFM, BRUKER Dimension Icon, Billerica, MA, USA) was conducted to evaluate the topography and surface roughness of the as-obtained TR membrane. Roughness measurements in this work were performed by surface analysis on a fixed area (5 × 5 μm^2^) taken from each sample.

### 2.4. Permeation Test

**Figure 2:**
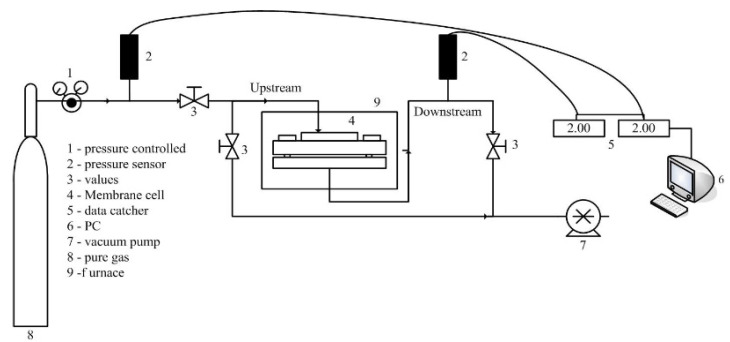
Schematic of gas permeation apparatus.

The pure gas permeation value of each as-fabricated TR membrane was measured by using lab-scale permeation measurement equipment, with an effective membrane area of 0.785 cm^2^ and an operation temperature of 28 ± 2 °C, by the constant volume/variable pressure approach (Figure 2). Before the permeation test, a vacuum pump was used to maintain the system in a vacuum state to avoid atmospheric gas interference. Afterwards, the membrane was mounted on a stainless-steel housing (Millipore Corp., Cat. No. XX4404700, Burlington, MA, USA) with a silicone O-ring. Two pressure transducers with a range of −1 to 9 bar (JPT-131S, Jetec Electronics Co., Ltd., Taichung City, Taiwan) were used to measure the feed and permeate pressures. A gas with a pressure of about 1.96 bar was fed into the upstream of the membrane and the downstream pressure, which increased with time (dp/dt), was recorded with a computer. Then, the gas separation performance of the membrane was obtained. To minimize the investigational error, each membrane sample was measured three times and the average value was reported. The detailed permeation process has been described in our previous work [21,22]. The tested gases in the present study contained hydrogen (2.89 Å), carbon dioxide (3.24 Å), oxygen (3.46 Å), nitrogen (3.64 Å), and methane (3.80 Å)—the values in parentheses correspond to the kinetic diameter. The study inferred the gas separation mechanism of the TR membrane based on the measured permeation results and related characterizations. Additionally, pure gas permeance was defined and calculated by Equation (3) and expressed according to the gas permeation unit (GPU = $1\times 10^{-6}\frac{cm^{3}\left(STP\right)}{cm^{2}\cdot s\cdot cmHg}$).

(3) $$P=\left[\frac{dp}{dt}\right]\frac{V\cdot T_{0}}{A\cdot \Delta p\cdot P_{0}}$$

where P represents the gas permeation value (GPU), *dp*/*dt* represents the increased rate of the downstream pressure at a steady state (cm Hg/s), *V* refers to the downstream volume (cm^3^), *T* is the permeation temperature (K), *A* is the effective area of the membrane (cm^2^), Δp represents the pressure difference between the feed side and the permeation side of the membrane (cm Hg)$,\;P_{0}$ is 76 cm Hg, and $T_{0}$ is 273 K.

The equation for selectivity calculation is as follows:

(4) $$\alpha_{i/j}=\frac{P_{i}}{P_{j}}$$

where $\alpha_{i/j}$ represents the ideal selectivity of pure gas i over j. $P_{i}$ and $P_{j}$ are the permeance value of gas i and gas j, individually.

## 3. Results and Discussion

### 3.1. Analysis of As-Received Rubber Sample

**Figure 3:**
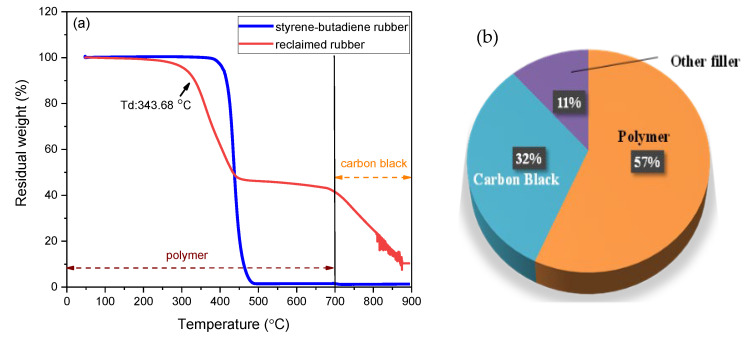
(**a**) TGA curve of commercial styrene–butadiene rubber and reclaimed rubber and (**b**) approximate weight composition of reclaimed rubber sample.

**Figure 4:**
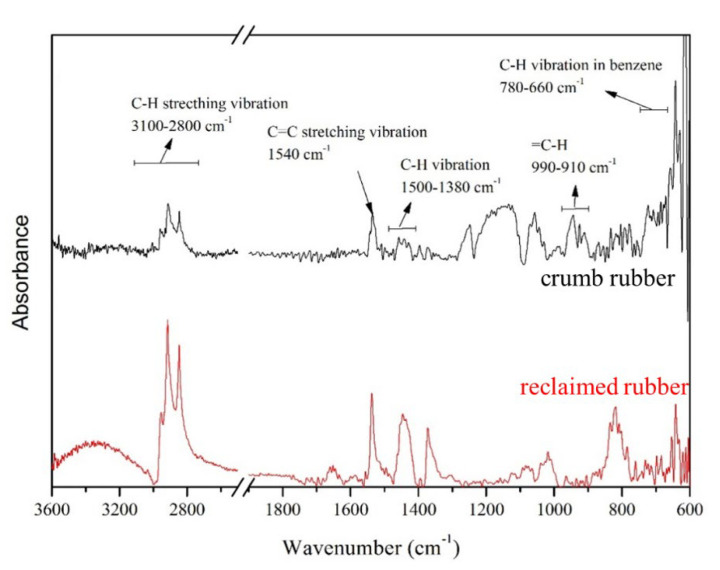
FTIR spectra of rubber samples before and after devulcanization.

The TGA analysis of commercial styrene–butadiene rubber (SBR) and reclaimed rubber were investigated by the TGA and their proximate compositions are shown in Figure 3. As expected, prior to heating to a temperature of 700 °C, commercial SBR suffered the greatest weight loss and finally decomposed completely at approximately 500 °C, which suggests that its whole composition consisted of polymer, that is, rubber. As presented in Figure 3a, for reclaimed rubber, the initial decomposition temperature (Td) was 343 °C and the weight-loss curve displayed a two-stage degradation process. The primary weight-loss was observed at approximately 350 °C, while the secondary weight loss was at approximately 750 °C, which implied at least two different compositions existing in the reclaimed rubber sample (i.e., polymer and carbon black). When the temperature reached 900 °C, approximately 11% of the sample weight remained, which was indicative of the presence of another filler. In addition, the initial decomposition temperature for reclaimed rubber is lower than that of commercial SBR, which suggests the presence of an impurity such as carbon black or another filler. As a result, based on the weight loss percentages corresponding to the degradation temperature, the approximate composition of reclaimed rubber was determined (Figure 3b). As anticipated, polymer accounted for 57% of the reclaimed rubber sample. Thus, using reclaimed rubber as a membrane precursor was feasible because of the abundant rubber polymer in the overall sample. Carbon black, which accounted for 32% of the reclaimed rubber, was the minor ingredient. A tire generally consists of 40–48% rubber polymer [13,14], and our findings show that the rubber polymer content is increased in reclaimed rubber. Additionally, a change in the functional group of the as-received rubber samples before and after devulcanization was detected using the FTIR technique to gain insight into the rubber sample properties (Figure 4). For the infrared spectroscopy of crumb rubber, the characteristic peaks at 3100–2800, 1540, 1500–1380, 990–910, 780–660, and 700–590 cm^−1^ were associated with the stretching vibration of C–H bonds, the stretching vibration of C=C bonds, C–H vibration, =C–H bands, aromatic C–H bonds, and C–S bonds, respectively [23]. The spectra for reclaimed rubber revealed that the characteristic peaks of C–H bonds and C=C bonds were increasingly apparent, and the intensity of C–S bonds was negligible, which mirrored the validity of the devulcanization procedure and validated the increase in the rubber polymer content of reclaimed rubber. In addition, the rubber type of the collected specimen from the local market was shown to be styrene–butadiene rubber because the detected functional groups of the rubber sample were similar to those of styrene–butadiene rubber from previous studies [24,25]. Due to the existence of carbon black and other fillers in the adopted precursor (reclaimed rubber), the light scattering degree would be affected by the black substance. Therefore, the typical peak of sulfur bridges is not easy to detect by the FTIR technique. As a result, the effect of devulcanization on reclaimed rubber was evaluated by the change in cross-linking density between rubber and reclaimed rubber. The cross-linking densities of both rubber samples, as reported in Table 1, were tested by the combination of the swelling method in the ASTM D6814 standard and the Flory–Rehner equation [19,20]. The devulcanization degree for reclaimed rubber, calculated by Equation (2), was 45.5%.

### 3.2. Effect of Reclaimed Rubber Precursor Concentrations on Gas Separation Performance

**Figure 5:**
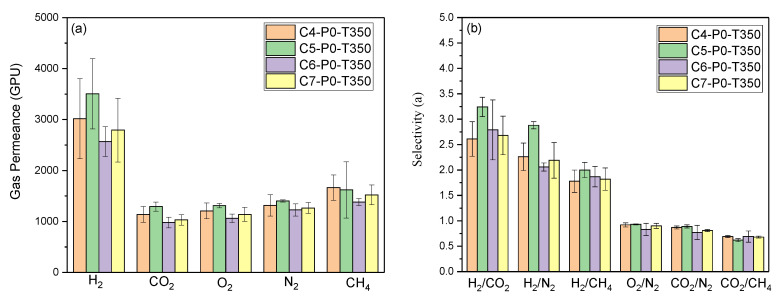
Gas separation performances obtained from resultant thermally rearranged (TR) membranes with different precursor concentrations. (**a**: single gas permeance; **b**: selectivity of different gas pairs).

**Figure 6:**
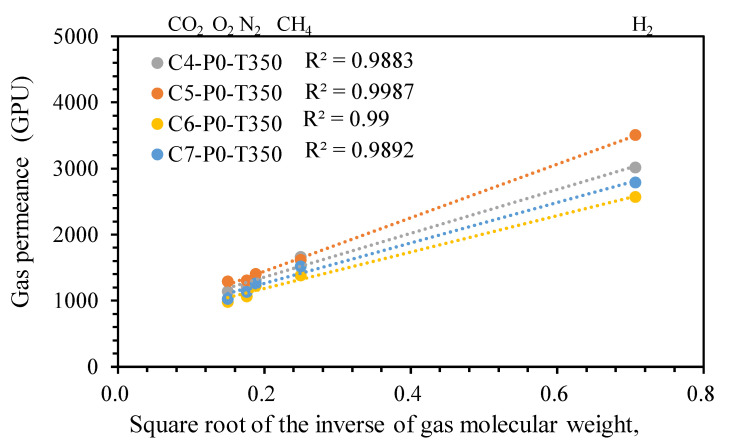
Single gas permeance of different gases obtained from resultant TR membranes with different precursor concentrations as a function of square root of inverse of gas molecular weights.

As shown in Figure 5, the gas permeance of all TR membranes with different precursor concentrations decreased in the following order: H_2_ (2.89 Å) > CH_4_ (3.80 Å) > N_2_ (3.64 Å) > O_2_ (3.46 Å) > CO_2_ (3.2 Å), where the values in parentheses represent the kinetic diameter. Evidently, the change in gas permeance for all rubber-derived TR membranes was uncorrelated with the kinetic diameter of tested gas. Moreover, all TR membranes exhibited higher selectivity over H_2_/CO_2_, H_2_/N_2_, and H_2_/CH_4_ separations than CO_2_/N_2_ and CO_2_/CH_4_ separation. The dominant gas separation mechanism for the membrane depends on the magnitude of the membrane pore. The Knudsen diffusion model, as a kind of gas transport mechanism, governs the gas separation process when the effective pore size of the membrane is smaller than the mean free path of penetrates, thus resulting in the linear relationship between gas permeance and the square root of the reciprocal gas molecular weight [26,27]. To further investigate the gas separation mechanism of rubber-derived TR membranes, we compared the gas permeance to the square root of the reciprocal gas molecular weight (Figure 6). As expected, correlations were observed between the gas permeance value and the square root of the reciprocal gas molecular weights for all TR membranes fabricated at different precursor concentrations (4–7 wt %). Their coefficients of determination, labeled R^2^, were in the range of 0.98–0.99. As a result, the predominant gas separation mechanism for all rubber-derived TR membranes was confirmed as Knudsen diffusion. The permeation of hydrogen of all rubber-derived membranes was higher than that of carbon dioxide, indicating that the rubber polymer was transformed into the unique microporous structure of TR membrane after thermal treatment due to the presence of aromatic chains in as-received reclaimed rubber (SBR confirmed in previous section), rather than dense rubber polymeric membrane. With regard to the lower experimental selectivity than theoretical values, this may be ascribed to the presence of surface diffusion because of the permeation temperature (28 ± 2 °C). The presence of a surface diffusion effect weakens the other mechanism (Knudsen diffusion).

### 3.3. Effect of Heating Temperature on Gas Separation Performance

**Figure 7:**
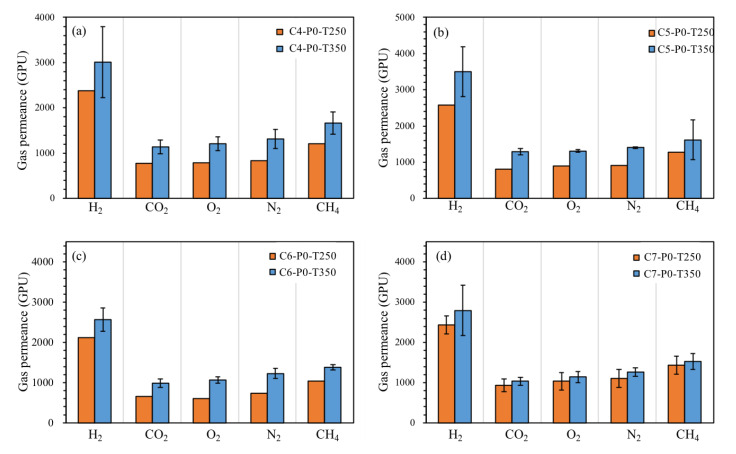
Single gas permeance of different gases obtained from resultant TR membranes with different heating temperatures. (**a**: 4 wt % precursor; **b**: 5 wt % precursor; **c**: 6 wt % precursor; **d**: 7 wt % precursor).

**Figure 8:**
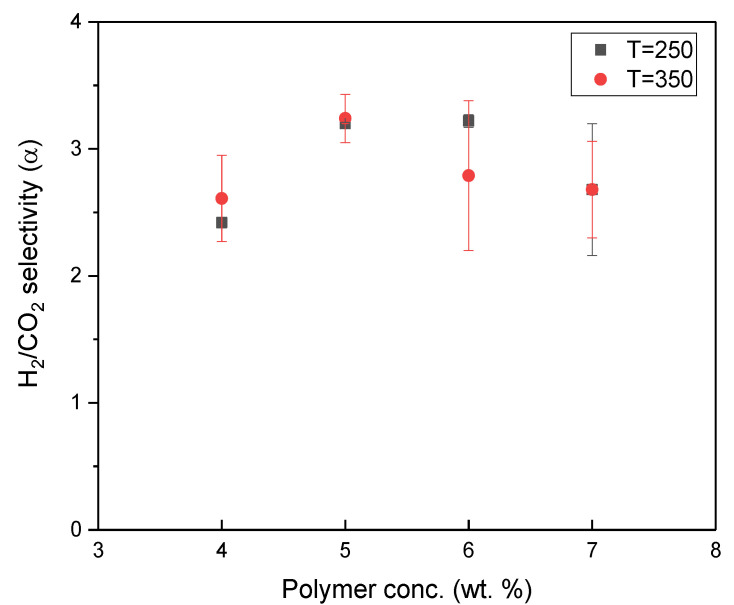
Selectivity over H_2_/CO_2_ gas pair of resultant TR membranes fabricated with different heating temperatures. (The icon in black represents the temperature at 250 °C; the icon in red represents the temperature at 350 °C).

The results of pure gas permeation obtained at different heating temperatures showed no apparent difference when compared to that obtained from a different precursor concentration, because the gas permeation order was the same (Figure 7). Consequently, the gas transport mechanism of TR membranes was still Knudsen diffusion when the heating temperature shifted from 350 to 250 °C. We also observed that the gas permeance of resultant membranes at a heating temperature of 350 °C, regardless of precursor concentration, were higher compared with membranes at a heating temperature of 250 °C. In the heating process at 350 °C, the bulking rings create more space to provide gases diffusion because of the higher thermal conversion; therefore, the effect of heating temperature on the as-prepared membrane is a crucial factor. In addition, the selectivity of the H_2_/CO_2_ gas pair obtained for all TR membranes as a function of precursor concentration and heating temperature is plotted in Figure 8. As shown in Figure 8, the selectivity of all membranes, regardless of heating temperature, was in the range of 2.4–3.5, which indicates that the influence of altering the precursor concentration or heating temperature on membrane permselectivity is insignificant. This infers that the pore size distinguishing between bigger gases (CO_2_) and smaller gases (H_2_) would not be substantially shrunk or enlarged with the changes in both parameters. The lower selectivity of the TR membrane was observed when the precursor concentration was too high (6 and 7 wt %); this could be correlated with the derived membrane thickness. Based on the work by D. Grosso [28], the degree of heat for the membrane surface and the membrane interior was uneven in the carbonization process and non-selectivity pores were produced as a result. Consequently, while the membrane was fabricated with higher concentration and higher heating temperature, a suppression in H_2_/CO_2_ selectivity was observed.

### 3.4. Effect of PPO Additive Content on Gas Separation Performance

**Figure 9:**
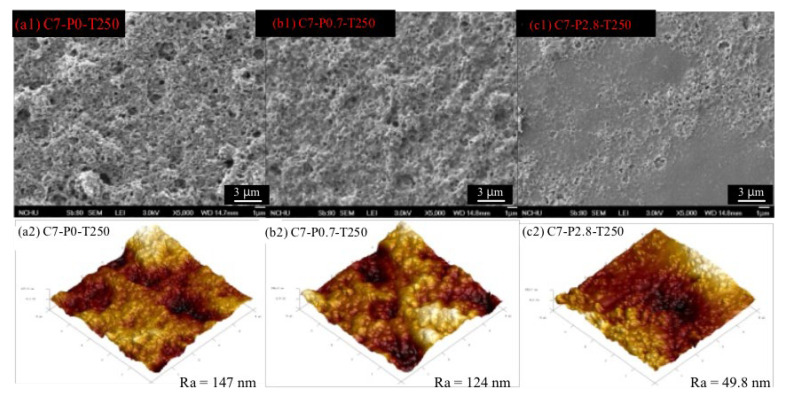
SEM images (**1**) of rubber-derived TR membranes as a function of polyphenylene oxide (PPO) additive content and corresponding atomic force microscopy (AFM) images (**2**). (**a**: C7-P0-T250; **b**: C7-P0.7-T250; **c**: C7-P2.8-T250).

Figure 9 shows the influence of incorporating PPO additive into the rubber precursor on the morphological structure of the derived TR membrane. No observable cracks were observed on the membrane surface of C7-P2.8-T250 compared with other TR membranes. Furthermore, the reduction of membrane surface roughness from 147 nm to 49.8 nm indicated a significant densification of the membrane surface after incorporating the PPO additive at 2.8 wt %. As expected, by adding the PPO modifier into membrane matrix, the membrane surface became smoother. The AFM results corroborated this finding, indicating that the membrane was successfully modified by the presence of PPO.

**Figure 10:**
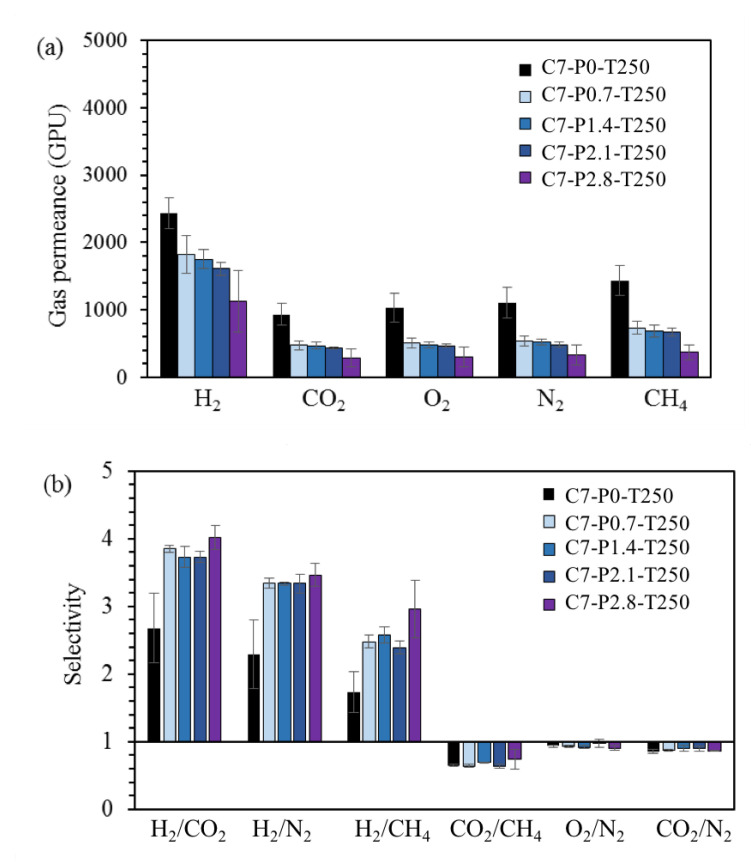
Gas separation performances obtained from rubber-derived TR membranes as a function of PPO additive content. (**a**: single gas permeance; **b**: selectivity of different gas pairs).

As shown in Figure 10, the C7-P2.8-T250 TR membrane exhibited the highest selectivity for all gas pairs. Specifically, H_2_/CO_2_ selectivity differed nearly by a factor of two and was observed to be 4 and 2.68 for C7-P2.8-T250 and C7-P0-T250, respectively. These findings indicate that the incorporation of PPO additive enhanced the permselectivity of the derived TR membrane for H_2_/CO_2_. These results can be attributed to the dense membrane structure obtained by blending a thermally stable polymer, in this case PPO, and are in accordance with previous studies [29,30]. According to the work of Ozaki [31], the pore structure of a carbon membrane is strongly influenced by polymeric precursors, and their thermal properties are vital in determining the derived carbon membrane performance. During thermal treatment, a thermally liable polymer precursor tends to decompose into volatile gas. This provides a diffusion path for gas, leading to an increase in permeance and a decrease in selectivity. The opposite trend in terms of gas permeance and selectivity was observed for the thermally stable polymer in this study. Hence, the addition of a thermally stable polymer (PPO, in this study) improved the resultant TR membrane’s selectivity because of the thermal stable property of the PPO during the heating procedure.

### 3.5. Comparison of Rubber-Derived TR Membrane with Other Published Literature

Table 2 compares the gas separation performance of the TR membrane derived from reclaimed rubber to that of TR membranes derived from expensive precursors [9,10,32,33,34,35]. The rubber-derived membrane at a lower thermal rearrangement temperature (250 °C) was comparable to other TR membranes fabricated with expensive polymer precursors at higher heating temperatures because of its acceptable selectivity, remarkable hydrogen permeability, and competitive and feasible commercial properties. As the hydrogen capacity of the demonstration plant in Japan is 50 Nm^3^/h [36], the plant needs a membrane with high flux but a reasonable separation factor for hydrogen enrichment. The C7-P2.8-T250 membrane synthesized during this work, with a hydrogen permeability of 1333 Barrer, exhibits a similar hydrogen permeance to membranes applied in a biogas plant [37]. Hence, the as-prepared membrane with high flux has potential for application in the commercial H_2_/CO_2_ gas-separation field. The binary gas mixture test will be performed in our future work to better understand the separation capability of rubber-derived membranes.

**Figure 11:**
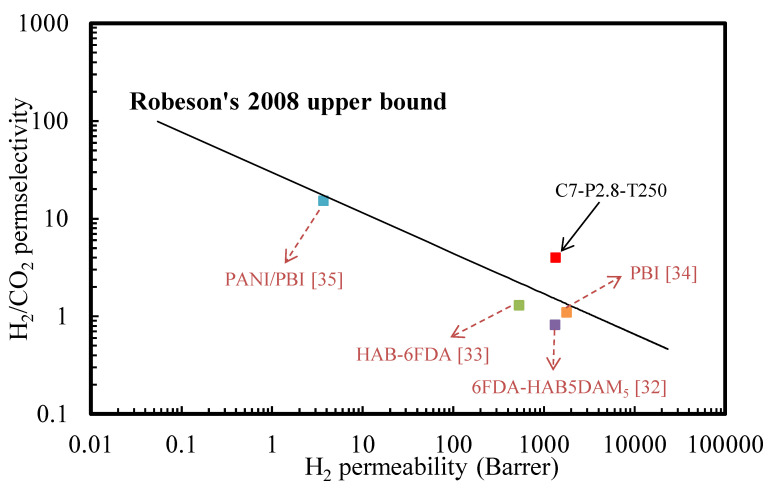
Robeson plot for H_2_/CO_2_ separation comparing the rubber-derived TR membrane with other TR membrane prepared by different precursors.

In addition, for membrane scientists, the Robeson upper bound set the appealing target to aim for and is a commonly used standard with which to evaluate the gas separation performance in the field of polymeric membranes. To compare our work with other studies in the field of TR membranes, the optimum gas separation result in this work (C7-P2.8-T250) and other published studies were illustrated in Robeson upper bound plots for H_2_/CO_2_ separation. The comparison clearly indicates that the gas transport capability of the rubber-derived membrane (C7-P2.8-T250) lies in the upper-right quadrant of Figure 11 and crosses the upper limit for H_2_/CO_2_ applications, showing that it can be recognized as an attractive candidate for H_2_/CO_2_ gas separation.

The practical feasibility for the membrane’s commercial application was preliminarily evaluated from the perspective of price per unit membrane area (USD/m^2^). The price per unit membrane area (USD/m^2^) was determined by the cost of the raw material, including the polymer precursor, and the used solvent and additive, etc. Table 3 summarizes the data and assumptions employed in this study. The cost of required solvent per ton rubber equals USD 50,309.4/ton, which was calculated from the solvent price multiplied by the rubber solubility in solvent. The cost of raw materials ranged from USD 51,864.40 to USD 52,975.40/ton, which was obtained by summing the price of reclaimed rubber [38] and the cost of required solvent and subtracting the value of national aid. The price per unit membrane area (USD/m^2^) in this work was calculated to be 2.1–3.2 USD/m^2^, which was acquired by multiplying the cost of raw materials and the required polymer per unit membrane. According to Richard W. Baker [39], if the adopted precursor is commercial polymer, these membrane modules vary in price from 10 to 100 (USD/m^2^), which is far more expensive than our figures. It is clear that the membrane prepared with reclaimed rubber is competitive in the capital market. We believe this technology is worthy of development in the future and could contribute to a significant reduction in tire waste.

## 4. Conclusions

Our main conclusions are as follows:

(a) The as-received reclaimed rubber was intensively investigated by TGA and FTIR analysis. The results revealed that the rubber type of reclaimed rubber is styrene–butadiene rubber, which accounted for more than half of the total components (57%).
(b) The presence of carbon black and other filler was also found, and their presence decreased the thermal stability of rubber.
(c) We measured the degree of devulcanization and the results showed that reclaimed rubber exhibited 45.5% devulcanization.
(d) According to the gas permeation tests, all rubber-derived TR membranes exhibited the Knudsen diffusion mechanism. In particular, the C7-P2.8-T250 membrane, with an ideal H_2_/CO_2_ selectivity of four and hydrogen permeability of 1333 Barrer, demonstrated a superior gas transport capability, mainly due to the conversion of the reclaimed rubber layer into a microporous membrane structure and the incorporation of a thermal stable filler.

In conclusion, these conditions showed that reclaimed rubber has significant potential as a precursor for fabricating TR membranes for the hydrogen enrichment industry. We have provided a viable approach to adopt reclaimed rubber as a precursor for preparing TR membranes with high performance for H_2_/CO_2_ separation.

## Figures and Tables

**Table 1:** Property of rubber samples before and after devulcanization.

Sample	Cross-Linking Density (10^−7^ mol/cm^3^)	Devulcanization (%)
Crumb Rubber	2.233	-
Reclaimed Rubber	1.217	45.50

**Table 2:** Comparison of rubber-derived TR membrane with other reports with respect to hydrogen permeability and H_2_/CO_2_ selectivity.

Polymer	Thermal Rearrangement Protocols	Permeability (Barrer) *		H_2_/CO_2_ Selectivity	Reference
		H_2_	CO_2_		
6FDA-HAB5DAM_5_	450 °C for 1 h	1318	1607	0.82	[32]
HAB-6FDA	450 °C for 1 h	530	410	1.29	[33]
Aromatic Polyimides	450 °C for 1 h	738	295	2.50	[9]
Poly(benzoxazole-co-imide)	400 °C for 2 h	222.1	172.8	1.29	[10]
Polybenzimidazole (PBI)	450 °C for 1 h	1779	1624	1.1	[34]
PANI/PBI	300 °C for 3 h	3.69	0.242	15.2	[35]
**C7-P2.8-T250**	250 °C for 2 h	1333	333.25	4.0	This work

*: Barrer = 1 × 10^−10^ cm^3^(STP)cm·cm^−2^ s^−1^ cmHg; the C7-P2.8-T250 membrane thickness is 1.116 μm.

**Table 3:** Summary of material price data and assumptions in this work.

Item	
The price of reclaimed rubber [38]	USD 1555–2666/ton
The required polymer per unit membrane	4–6 × 10^−5^ ton/m^2^
The price of solvent (toluene)	USD 0.52/100 mL
The rubber solubility in solvent	1.04 × 10^−5^ ton reclaimed/100 mL solvent
National aid	USD 100/ton reclaimed rubber
